# Prion Positivity Detected by Real-Time Quaking-Induced Conversion (RT-QuIC) in the Cadaver of an Elderly Woman Subjected to Forensic Autopsy

**DOI:** 10.7759/cureus.78199

**Published:** 2025-01-29

**Authors:** Takehiro Nakagaki, Takahito Hayashi, Miho Kaneko, Akio Akagi, Bungo Furusato, Yasushi Iwasaki, Katsuya Satoh, Kazuya Ikematsu, Noriyuki Nishida

**Affiliations:** 1 Department of Molecular Microbiology and Immunology, Graduate School of Biomedical Sciences, Nagasaki University, Nagasaki, JPN; 2 Department of Legal Medicine, Graduate School of Medical and Dental Sciences, Kagoshima University, Kagoshima, JPN; 3 Department of Neuropathology, Institute for Medical Science of Aging, Aichi Medical University, Nagakute, JPN; 4 Clinical Genomics Center, Nagasaki University Hospital, Nagasaki, JPN; 5 Department of Health Sciences, Unit of Medical and Dental Sciences, Graduate School of Biomedical Sciences, Nagasaki University, Nagasaki, JPN; 6 Department of Forensic Pathology and Science, Unit of Medical and Dental Sciences, Graduate School of Biomedical Sciences, Nagasaki University, Nagasaki, JPN

**Keywords:** abnormal prion protein, forensic autopsy, prion diseases, rt-quic, undiagnosed case

## Abstract

Prion diseases are fatal neurodegenerative disorders. Previous studies have indicated the presence of "prion carriers" who remain asymptomatic, but scrapie prion protein (PrP^Sc^) has begun to accumulate in the brain. Indeed, we identified an undiagnosed case of prion disease in a cadaver used for the anatomical practice of medical students. These findings suggest that cadavers for autopsy may occasionally include prion carriers. In the case of forensic autopsy, staff cannot sometimes obtain the background information of the dead bodies, and the risks for exposure to prions can be higher than in other autopsies. To ensure the safety of forensic staff, we conducted prion screening tests on the brains of the cadavers. One case demonstrated positive results in the real-time quaking-induced conversion (RT-QuIC) assay, which amplifies abnormal prion proteins in vitro. This result indicates that asymptomatic cases exist not only in cadavers but also in living individuals. The risk of prion infection via medical procedures including autopsy is not high, but prions are lethal pathogens that are difficult to decontaminate. Medical staff should consider that the cadaver or patient can be a prion carrier regardless of whether they are symptomatic or asymptomatic. This type of prion investigation allows us to obtain information on pre-symptomatic cases of prion disease, which could contribute to enhanced medical safety and provide new insights into human prion diseases.

## Introduction

Human prion diseases are classified as sporadic, genetic (familial), and acquired. They are characterized by the accumulation of abnormal prion protein (PrP) (also called scrapie prion protein (PrP^Sc^)), gliosis, and spongiform changes in the brain [1]. Once PrP^Sc^ is generated or enters the central nervous system, it gradually expands in the brain. Based on cases of acquired prion diseases, the incubation period from the initial accumulation of PrP^Sc^ to the onset of symptoms is sometimes more than 10 years [2]. This finding indicates that asymptomatic individuals with PrP^Sc^ in the brain (referred to as "prion carriers") can exist. Indeed, two cases of prion transmission via organ transplantation from prion carrier donors have been reported [3,4]. We previously identified an undiagnosed case of prion disease in a cadaver used for anatomical practice [5]. This finding suggests that prion carriers may exist even among dead bodies for autopsies. Compared to anatomical practices, forensic autopsies are conducted more frequently and often lack detailed information about the deceased's medical history. In light of this, we have initiated screening tests on cadavers during forensic autopsies and report the finding of a prion-positive case.

## Case presentation

Methods

Preparation of Brain Homogenates (BH)

During the autopsy, two samples each from the frontal lobe and temporal lobe of each cadaver, totaling four samples, were collected and frozen. One fragment from the frontal lobe and one from the temporal lobe were weighed, and phosphate-buffered saline (PBS) was added to make a 10% w/v solution. The samples were homogenized using a multi-bead shocker (Yasui Kikai, Osaka, Japan) and stored at -80°C [6].

Screening Test 

The screening tests were performed using the real-time quaking-induced conversion (RT-QuIC) method, which detects PrP^Sc^ from a small amount of tissue by promoting the conformational conversion of recombinant PrP through shaking. The methods for the expression and purification of recombinant human PrP (residues 23-231) have been previously described [7,8]. The BH containing 10^-6^ g of brain tissue and the reaction buffer which is composed of 500 mM sodium chloride (NaCl), 50 mM piperazine-N,N′-bis(2-ethanesulfonic acid) (PIPES) (pH 7.0), 1 mM ethylenediaminetetraacetic acid (EDTA), 0.001% sodium dodecyl sulfate (SDS), 0.01 mM thioflavin T (ThT), and 0.1 mg/ml recombinant human PrP (residues 23-231) were loaded into a 96-well plate with a clear bottom (Greiner, Frickenhausen, Germany). Four wells were used for each sample (four replicates for each). The plate was sealed (plate sealer, Nalgene Nunc International, Rochester, NY, USA) and incubated at 37°C in a FLUOstar OMEGA (BMG LABTECH, Ortenberg, Germany) with cycles of 60 seconds shaking (1000 rpm double orbital) and 60 seconds incubation. ThT fluorescence was measured every nine cycles (450 ± 10 nm excitation and 480 ± 10 nm emission; bottom-read) for a total of 150 times.

Confirmation Test

The specimens that tested positive in the screening test were further analyzed using the RT-QuIC method. For the confirmation test, considering the possibility of specimen contamination during the screening test, new specimens that had been frozen were homogenized for use in the test. The BH was serially diluted, and six wells were used per specimen (six replicates). The RT-QuIC conditions (such as rotation speed, reaction temperature, reaction buffer, and recombinant PrP) were identical to those used in the screening test. The 50% seeding dose (SD_50_) was calculated using the Spearman-Kärber method [8].

Sequencing Analysis of the Coding Region of Prion Protein Gene (PRNP)

Genomic DNA was extracted from 25 μg of frozen liver tissue from the patient using NucleoSpin Tissue® (Takara Bio, Shiga, Japan) according to the manufacturer's instructions. The coding region of PRNP was amplified by polymerase chain reaction (PCR) using PrimeSTAR GXL DNA Polymerase (Takara Bio) and gene-specific primers (5’ tcctcttcattttgcagagcagtc 3’ and 5’ gcctccctcaagctggaaaaag 3’). The amount of template used was 500 ng of the purified DNA. After the purification of the PCR product, the nucleotide sequences were determined by the Sanger method with ABI PRISM 3130xl Genetic Analyzer (Thermo Fisher Scientific, Waltham, MA, USA) and BigDye Terminator Cycle Sequencing Ready Reaction kit, version 3.1 (Thermo Fisher Scientific).

Pathological Analysis

The paraffin blocks were cut at a thickness of 4.5 μm for immunohistochemistry and 9 μm for hematoxylin-eosin staining. Immunohistochemical analysis was carried out with an anti-PrP antibody (3F4 (Dako, Glostrup, Denmark), mouse monoclonal, diluted 1:100) after hydrolytic autoclaving for antigen retrieval. Immune complexes were visualized with a standard avidin-biotin method using Histofine Simple Stain Max PO (Nichirei, Tokyo, Japan) and 3,3’-diaminobenzidine chromogen (DAB, Wako Pure Chemical Industries, Tokyo, Japan). Immunostained sections were lightly counterstained with Mayer's hematoxylin.

Results

Case Profile

The deceased was a woman in her early 80s living with her son in his early 50s. Until two years ago, she had visited the hospital regularly for hypertension, but after experiencing anaphylactic shock caused by a prescription drug, she began to distrust medical institutions and refused to go to the hospital. According to her son, the deceased showed no signs of dementia. One day in October, her son found her dead in the supine position on a futon laid out in her bedroom. According to her son, about a week before her death, she suddenly lost her appetite, and the day before she died, she did not ask for water.

Autopsy Findings

The deceased was 140.5 cm tall and weighed 22.1 kg (BMI 11.2), with a subcutaneous adipose tissue thickness of 1.0 cm at the umbilical region and rather poor fat deposition in the large omentum. A shallow pressure ulcer was observed on the right inner side of the sacral region (1.5 × 1.0 cm) and midline of the sacral region (0.8 × 0.5 cm). There were no major injuries on the whole body except for a few crusted, broken epidermal abrasions on the medial side of the left scapular region. The heart weighed 205 g with moderate atherosclerosis in the left coronary artery and mild atherosclerosis in the right coronary artery, but no hemorrhage or scarring in the myocardium. The heart contained dark red fluid blood with soft clots. The brain is slightly atrophic at 1025 g, but no spongiform changes were observed. The right and left lungs (left 175 g, right 280 g) were dry and collapsed with no edema. Other organs were generally dry, with no findings suggestive of advanced disease. Histopathological examination revealed no spongiform changes, kuru plaques, or accumulation of PrP^Sc^ in the brain. No drug toxicants, including alcohol, were detected in the blood or urine collected during the autopsy. Blood biochemistry tests showed C-reactive protein (CRP) of 0.157 mg/dl (reference value: <0.14 mg/dl), acetone of 134 mg/ml (reference value: <5 mg/ml), and ketone fractions of 158 mmol/l for acetoacetic acid (reference value: <55 mmol/l) and 4223 mmol/l for 3-hydroxybutyric acid (reference value: <85 mmol/l).

Based on the above autopsy findings, the various laboratory findings, and a comprehensive assessment of the corpse's preexisting conditions, the cause of death was diagnosed as acute circulatory failure due to dehydration.

Detection of Abnormal PrP

**Figure 1:**
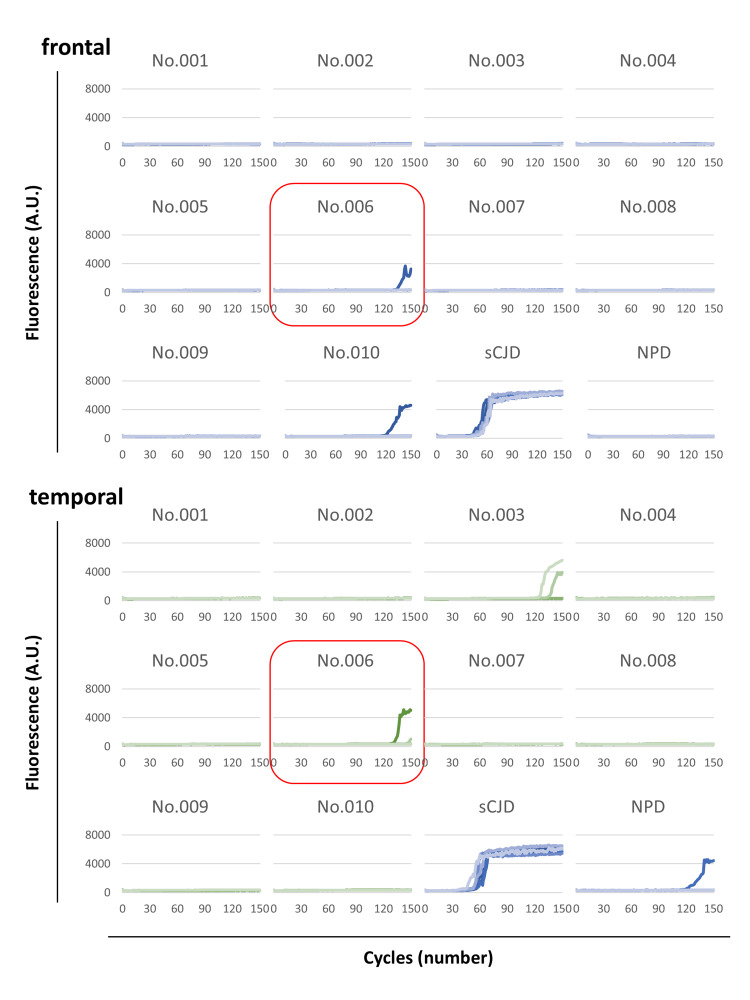
The screening tests of cadavers for forensic autopsy. The homogenized frontal and temporal lobes were utilized as seeds for prion screening using the RT-QuIC method. Upon the reaction with abnormal PrP, recombinant PrP is converted to a beta-sheet-rich structure, forming amyloid. The reaction has been induced by shaking. ThT in the reaction buffer binds to amyloid and its fluorescence increases. Brain homogenates from patients with sCJD and non-prion disease were used as the positive and negative controls, respectively. The vertical axis shows the fluorescence value, and the horizontal axis shows the number of detection cycles. One microgram (10^-6^ grams) of brain tissue was used as the seed for each well. Each sample was replicated four times (four wells per sample). This figure presents a subset of the screening test results. Samples No.001 to No.010 correspond to individual sample numbers. Positive signals were observed in specimens of both the frontal and temporal lobes of No.006. RT-QuIC: real-time quaking-induced conversion; ThT: thioflavin T; sCJD: sporadic Creutzfeldt-Jakob disease; AU: arbitrary unit; NPD: non-prion disease

The samples were collected from two distinct sites in each of the frontal and temporal lobes of the brain during autopsy and stored at -80°C. One specimen of each lobe was homogenized for the screening test, and the others had been stored at -80°C until additional tests were performed. Screening tests were performed using the RT-QuIC method [9] with wild-type human recombinant PrP (23-231) as a substrate. A positive signal was detected in one out of four wells of each specimen taken from the frontal and temporal lobes of the cadaver (No.006) (Figure 1). The frontal lobe was retested, and a positive signal was again detected, so No.006 was determined to be screening positive. Some of the other cadavers also showed positive signals in the screening test (such as No.003 and No.010 in Figure 1), but these were observed to have positive signals only in the frontal lobe or the temporal lobe. When retested, these also turned negative, so they were considered screening negative.

**Figure 2:**
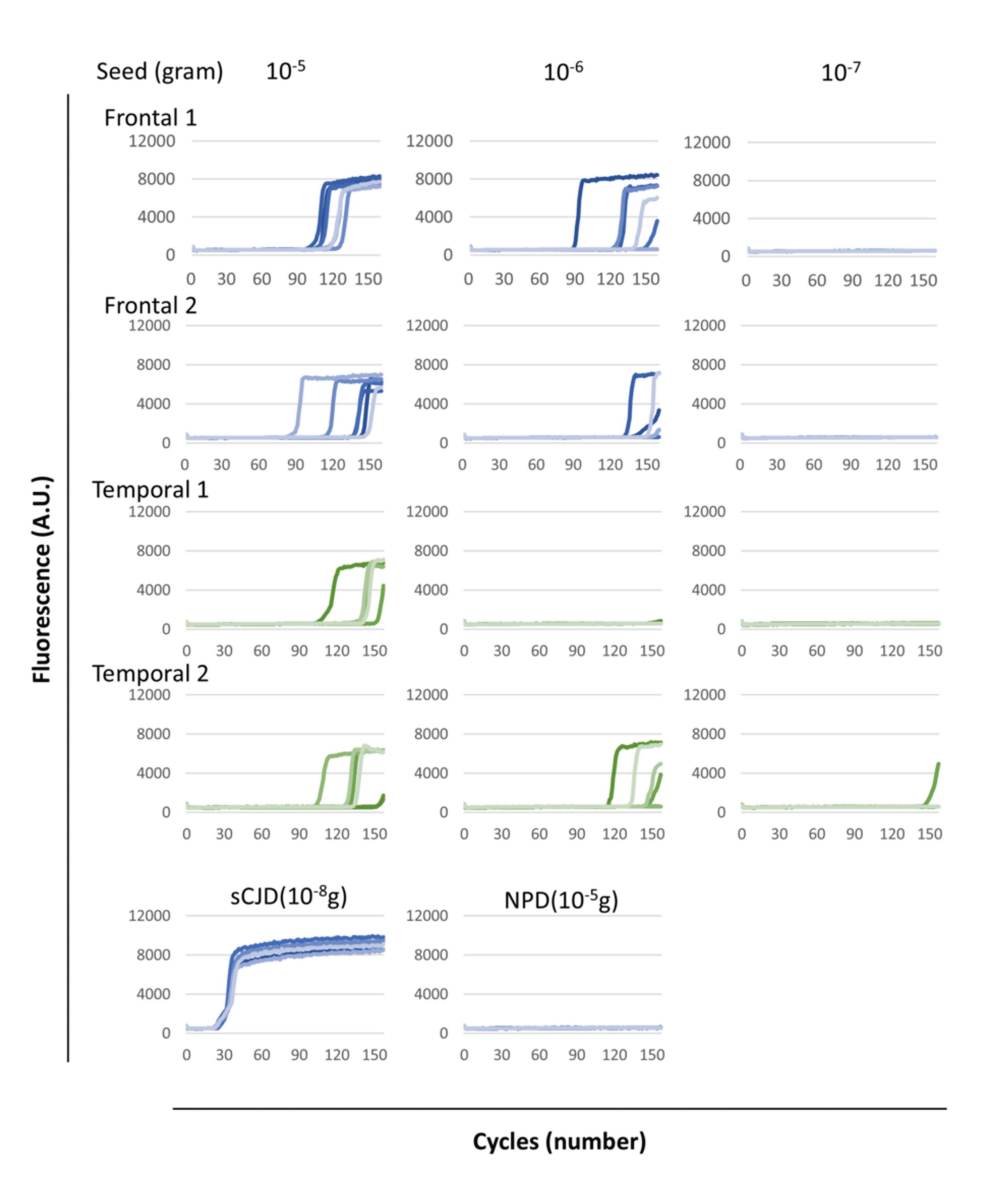
Confirmatory examinations using the RT-QuIC method. The samples from the frontal and temporal lobes of the cadaver were used as seeds of RT-QuIC. The amounts of brain tissues used as seed for each well are shown above the graph. Each sample was replicated six times (six wells per each sample). Positive signals were observed in all specimens. RT-QuIC: real-time quaking-induced conversion; NPD: non-prion disease; sCJD: sporadic Creutzfeldt-Jakob disease

Then, one specimen of each of the frontal and temporal lobes, which had been kept for additional tests, was further split into two pieces each and examined to confirm whether the cadaver was truly prion positive. Positive signals were observed in all wells containing 10^-5^ g of brain tissue from the frontal lobe. Additionally, four or more out of the six wells with 10^-5^ g of tissue from the temporal lobe were positive (Figure 2).

The SD_50_ of each sample were calculated using the Spearman-Kärber method. The log SD_50_ per gram of brain tissue for Frontal 1, Frontal 2, and Temporal 1 were 6, 6.17, and 5.5, respectively. SD_50_ could not be determined for Temporal 2 because no dilution concentration resulted in 100% positivity. Given that the log SD_50_ of the positive control (sporadic Creutzfeldt-Jakob disease (sCJD)) in Figure 2 was 10.0, it is considered that the amount of PrP^Sc^ in this individual's brain was extremely low.

DNA Sequencing Analysis and Histopathological Findings

**Figure 3:**
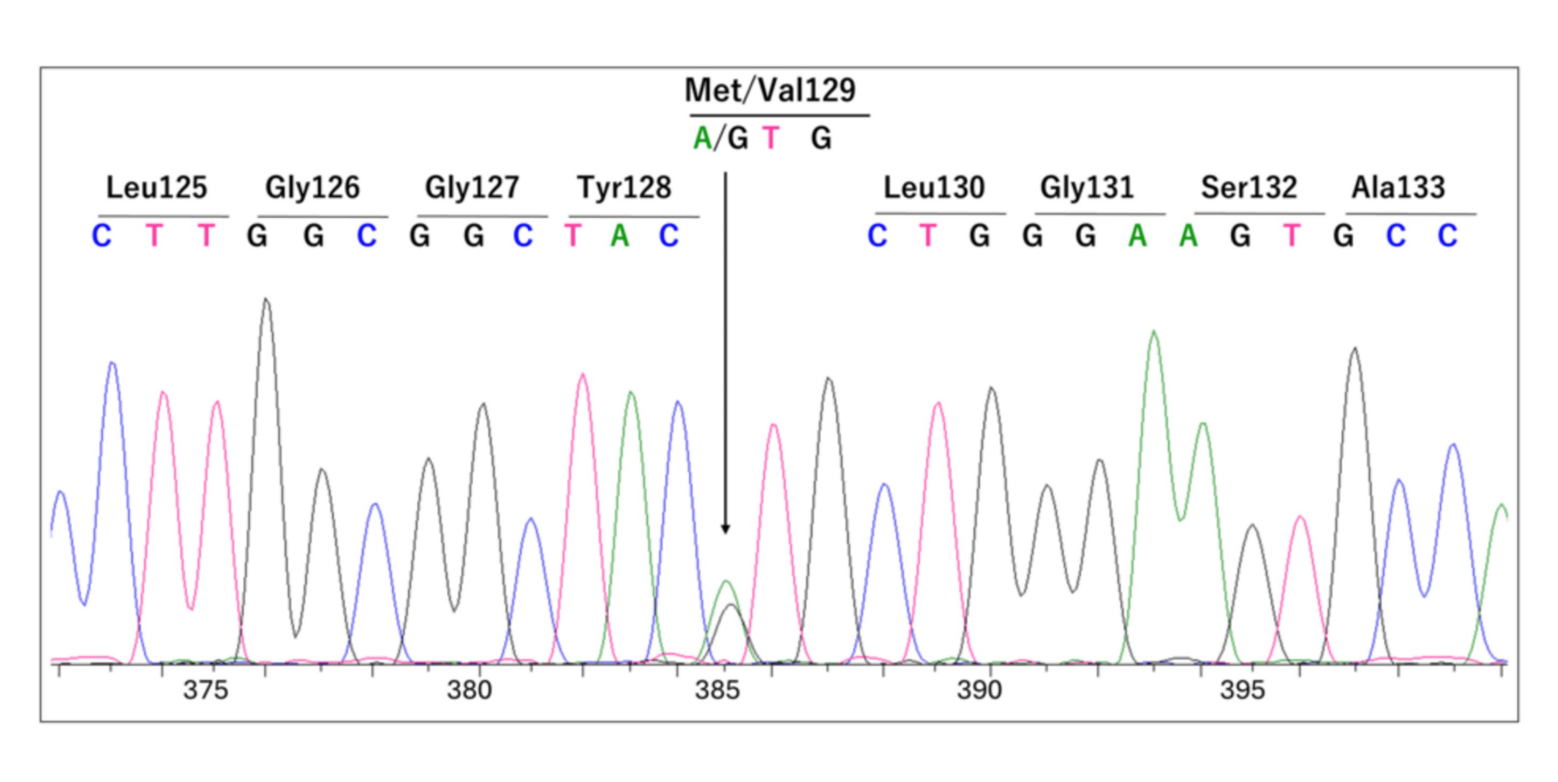
Sanger sequencing result of the cadaver's prion protein gene. Part of the result of sequencing analysis of amplified DNA. Double peaks were observed at nucleotide position 385. This represents the heterozygosity of "A" (green peak) and "G" (black peak). They are translated into the amino acids methionine and valine at codon 129.

Sanger sequencing of the PRNP of the cadaver revealed two peaks corresponding to adenine and guanine at nucleotide position 385 in the protein-coding region (Figure 3).

**Figure 4:**
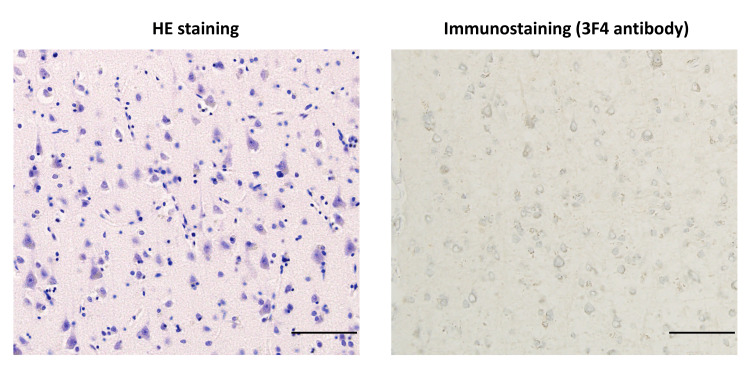
Histopathological analysis of the brain. Microscopic examination of the brain. HE staining (left) and immunohistochemical staining for abnormal prion protein (right) in the frontal cortex. No spongiform changes, kuru plaques, or PrP^Sc^ accumulation was observed. The scale bars indicate 50 μm. HE: hematoxylin and eosin; PrP^Sc^: scrapie prion protein

This indicates heterozygosity for methionine and valine at codon 129. Furthermore, no mutations associated with genetic prion disease were detected. Histopathological examination revealed no spongiform changes, kuru plaques, or PrP^Sc ^accumulation (Figure 4). 

## Discussion

The results of the DNA sequencing analysis indicated the possibility of sCJD. Because supporting data other than RT-QuIC are lacking, it might be difficult to diagnose this cadaver as a prion disease. However, the fact that RT-QuIC can detect PrP^Sc^ at the femtogram (10^-15^ g) level raises the possibility that this case was at a very early stage of the disease [7]. The amount of accumulated PrP^Sc^ can vary by region, even within the frontal lobe, especially during the early stages of the disease. This might account for the difference in the reactivity of RT-QuIC between screening and confirmatory tests.

Due to the low amount of PrP^Sc^ detected in this cadaver, the risk of transmission is likely minimal. However, this case highlights that latent sCJD can be present not only in cadavers but also in living individuals. Furthermore, it cannot be ruled out that latent CJD cases with substantial PrP^Sc^ accumulation may undergo medical procedures, posing a potential risk. In addition, PrP^Sc^ is not completely inactivated by general methods such as autoclaving (121°C for 15 minutes) or formalin disinfection.

Prion diseases are rare, with a global incidence rate of 1-2 cases per million population annually. Meanwhile, the incidence of CJD in Japan, with the most advanced aging population, was reported more than 4.5 per million in 2014 [10]. Additionally, the incidence of sCJD, but not genetic CJD, is also rising worldwide, according to a report from the Creutzfeldt-Jakob Disease International Surveillance Network [11]. Based on these reports, the increase in the incidence of prion diseases is likely attributable to population aging. We have found two positive cases in the screening tests, including this report, in the past two years [5]. It is conceivable that there may be a significantly larger number of asymptomatic cases in sporadic and genetic prion diseases compared to the number of diagnosed patients. For these reasons, medical staff should consider the possibility of latent prion disease [5].

The purpose of this screening is to ensure the safety of staff and students. However, its continued implementation is expected to accumulate valuable data on the frequency and pathological features of pre-symptomatic cases of human prion diseases. This could contribute to elucidating the pathological changes associated with prion diseases and enhancing the safety of medical practices.

## Conclusions

Although prion diseases are rare, the number of carriers may exceed the estimated prevalence. It is important to consider the possibility that individuals in medical settings could be prion carriers, regardless of whether symptoms are present. Strict adherence to standard precautions is essential to prevent prion contamination. This screening may contribute to gathering valuable information on prion carriers.
